# Beyond empathy decline: Do the barriers to compassion change across medical training?

**DOI:** 10.1007/s10459-022-10100-2

**Published:** 2022-04-07

**Authors:** Clair X. Y. Wang, Alina Pavlova, Antonio T. Fernando, Nathan S. Consedine

**Affiliations:** 1 Department of Psychological Medicine, University of Auckland, Building 507, Room 3008, Auckland, New Zealand; 2 Faculty of Medical and Health Sciences, The University of Auckland, Private Bag 92019, Auckland, New Zealand

**Keywords:** Health, Healthcare, Compassion, Empathy, Medical students, Clinical training, Self-compassion

## Abstract

Background: Despite being a mandated, foundational value in healthcare, research on compassion remains limited. Studying the individual, patient, clinical, and contextual factors that interfere with compassion—the “barriers”—may clarify our understanding of the origins of compassion and identify potential targets for improving patient-centred care. Studies of the related construct of empathy have suggested that medical students report declines with increasing clinical experience. In contrast, when comparing physicians with medical students, increased clinical experience predicts lower barriers to compassion. Whether—and how—a similar experience-related decline in the factors that interfere with compassion occurs across medical training remains unknown.

Aims: To describe how the barriers to compassion vary across clinical training in medical students.

Method: New Zealand medical students (N = 351) in their clinical years (Years 4–6) completed measures of the Barriers to Physician Compassion (BPCQ) and potential covariates such as demographics, work burden factors, and dispositional factors. The BPCQ indexes the extent to which barriers in four domains (individual, patient, clinical, and contextual) interfere with a physician/student’s compassion towards patients. Analyses of variance and regression analyses were used to explore the effect of year level on the four types of barriers.

Results: Year 4 students reported slightly lower student-related, environmental and patient/family-related (but not clinical) barriers than Year 6 students (effect size: ɷ^2^ < 0.05); all barriers increased comparably across training. Controlling for relevant confounds, regression analyses confirmed that lower year level predicted lower barriers to compassion. Higher self-compassion, but not gender, predicted lower barriers.

Conclusions: In extending studies of empathy decline, this report suggests that students experience higher barriers to compassion as clinical training progresses. This is in contrast to existing studies contrasting physicians with medical students, where greater experience was associated with lower perceived barriers to compassion. Self-compassion may offset increases in barriers to care.

## Introduction

Healthcare practitioners, including medical students, are expected to demonstrate “compassionate treatment of patients” (American Medical Association, 2016; Department of Heath & Social Care, 2015; MSOP Report Writing Group et al., 1998; New Zealand Medical Association, 2020; World Medical Association, 2018). While the study of compassion in medicine has been hampered by the lack of a unified definition and appropriate measures (Gu et al., 2019; Sinclair et al., 2017), reports of a lack of compassion in healthcare(Francis, 2013) have placed increasing attention on this topic in recent years. New research outlining the factors predicting prosocial traits and motivations in physicians, such as empathy and compassion, is continuously emerging (Pavlova et al., 2021). Yet, little is known about the factors that predict compassion (or a lack thereof) in medical students and how these develop over time.

The Transactional Model of Compassion suggests that factors which influence compassion can be grouped into four domains—factors related to the *physician*, the *patient* (and/or their family), *clinical issues* (i.e. related to the medical situation), and the *environment* (e.g. physical, institutional, policy) (Fernando & Consedine, 2014a, 2014b)*.* Of relevance to this paper, the model can also be applied when thinking of factors which interfere with compassion i.e. the *barriers* to compassion (Fernando & Consedine, 2014b). Prior studies have suggested that the barriers to compassion tend to *decline* with greater clinical experience and age among healthcare professionals (i.e. nurses, physicians and medical students) (Dev et al., 2019; Fernando & Consedine, 2017). Moreover, physician-related, environmental and clinical barriers are lower in physicians compared to medical students (Dev et al., 2019). In extending this prior work on how the barriers to compassion differ across medical training, the current paper focuses on how the barriers vary by year level *among* medical students.

The suggestion that prosocial processes, or the predictors of prosocial processes, vary over the course of medical education is not new. The study of empathy—“an uncritical understanding of a patient’s inner feelings and experiences as a separate individual” (Hojat et al., 2001, p. 352)—has also shown variations with clinical experience. Among medical students—albeit with some exceptions (Ferreira-Valente et al., 2017; Quince et al., 2016; Smith et al., 2017)—empathy has generally been shown to decline by year of study (Diseker & Michielutte, 1981; Lim et al., 2013; Park et al., 2015; Spatoula et al., 2019). Although empathy and compassion are distinct constructs (Strauss et al., 2016), this observed “empathy erosion” over the course of medical school is notably in contrast to the finding above, where the barriers to compassion are *lower* with increasing experience. This paper attempts to address this contradiction by understanding the trajectory of the barriers to compassion in medical students and how these might compare to the reasons behind empathy decline.

In trying to understand the origins of a possible decline in empathy across training, two consistent interpretations have emerged in the literature. First, it has been suggested that pressures from the clinical environment and/or a “hidden curriculum” are responsible. As trainees, students likely feel pressured to adapt their behaviour to match that of superiors and the culture of their clinical environments, which may be problematic when there is an absence of empathic role models or where senior colleagues demonstrate negative behaviour and attitudes (Tavakol et al., 2012). The value placed on biomedical and evidence-based medical practice in assessments and clinical practice, sometimes at the expense of humanistic care, may also foster the impression that biomedical knowledge is more important than humanistic qualities, leading students to sacrifice empathy for objectivity (Mahoney et al., 2016; Perrella et al., 2019; Wright et al., 2019). Such dynamics (i.e., emphasising objectivity at the expense of empathy) may be compounded by external pressures such as high workloads (Mahoney et al., 2016), long shifts (Ren et al., 2016), and a lack of sleep (Hojat et al., 2009).

Second, studies also support the view that empathy may decline as a result of the internal psychological changes necessitated when adjusting to these new environmental factors (e.g., defence mechanisms, coping skills etc.). Most obviously, such internal psychological changes would include the processes related to the need to protect the self against distress and burnout (Mahoney et al., 2016; Neumann et al., 2011). While junior trainees may approach clinical placements with strong ideals (Gaufberg et al., 2010), they also report a fear of becoming (and can become) overwhelmed if they are too empathic (Neumann et al., 2011). Across training, students may also begin to identify more as doctors and less as members of the public, creating an “in-group/out-group” dynamic in relation to their patients (Triffaux et al., 2019).

In investigating how the barriers to compassion vary by year of medical education, the findings from this research could further our understanding of whether, similar to the reasons behind empathy decline, particular barriers to compassion increase as students grapple with the realities of clinical practice. Given the reasons thought to contribute to empathy decline (e.g. fatigue, emotional exhaustion, the need for self-protection) also feature in qualitative studies investigating compassion in medical students (Tierney et al., 2018; Wear & Zarconi, 2008), increases in the barriers to care seem likely. Conversely, if we are to confirm that there is an experience-related decline in (certain) barriers to compassion for medical students across clinical training, this may point to further differences between empathy and compassion, which may have implications for differential training needs.

Thus, the broadest aim of the current report was to assess whether there are global differences in students’ experiences of barriers to compassion across different stages of clinical medical education. A secondary, more exploratory question regarded whether different *types* of barriers (physician (student)-related, patient-related, clinical, or environmental barriers) vary to the same degree. Evaluating whether different barriers appear to be changing at the same rate is important because it may help illuminate the *origins* of changes in prosociality across time. Hypothetically, if compassion is influenced by the same dynamics that impact empathy (i.e. a combination of environmental influences and internal processes), we might expect to see *generally* higher barriers to compassion as students’ progress through their medical education. However, it might also be that *specific types* of barriers increase more rapidly across training, which will require extra attention and specific interventions.

## Methods

### Study setting and design

This study is part of a large-scale survey project investigating compassion in healthcare (Dev et al., 2018, 2019, 2020), approved by the University of Auckland Human Participants Ethics Committee (Approval Number: 7640). Data were collected via non-random convenience sampling during the 2013–2014 calendar years with healthcare practitioners (i.e., nurses, physicians, and medical students) accessed via a lecture series, medical meetings and hospital grand rounds, contacts and referrals in hospitals and clinics in New Zealand, and e-mails distributed via professional organizations (i.e., District Health Boards, Primary Health Organizations, speciality interest groups, and medical schools). Inclusion criteria required that participants were practicing as medical professionals, were registered nurses, or were enrolled as medical students in New Zealand and were seeing patients during the past 12 months of their training. After providing informed consent, a 20-min online survey was administered. Participation was voluntary and anonymous.

We acknowledge that the same individual may have received the survey twice had they been on two different mailing lists. However, it is unlikely that an individual would complete the same survey twice during the same year, because the Qualtrics submission had a “prevent multiple submissions” condition in place that restricts one’s IP address to a single submission via an anonymous link that has been sent during this year.

### Participants

Of the 1700 participants in the parent project, 351 were medical students in their clinical years of training (i.e. 4th to 6th year) from both universities offering medical programmes in New Zealand (the Universities of Auckland and Otago). Students at both universities undergo a six-year degree, with three years of pre-clinical training (i.e. lecture-based learning; reduced to two if students have a undergraduate degree) followed by three years of clinical training (i.e. full-time clinical placements). Although the data analysed in this study were collected some time ago (2013–2014), no major curricular changes have taken place in New Zealand medical education in the intervening time with regard to compassion education. Thus, while it is possible, the chances of major (unmeasured) differences in curricula or student cohort composition across this time seem unlikely.

We excluded medical students in pre-clinical training (i.e. years one to three) given concerns about the validity of ratings in the absence of clinical experience. The final sample consisted of 104 fourth-year students (66.3% female, n = 69), 111 fifth-year students (65.8% female, n = 73), and 136 sixth/final-year students (58.8% female, n = 80), representing 19.4% of the clinical medical student population at the time (New Zealand MSOD Steering Group et al., 2018). A comparison of the sociodemographic characteristics of the study sample and the population of New Zealand medical students graduating around this time period is provided in Table 1 (New Zealand MSOD Steering Group et al., 2017).

**Table 1:** Comparison of study sample (N = 351) to national population of medical students graduating in 2012–2014

	Our study sample (N = 351)	National MSOD report for graduating classes of 2012–2014 New Zealand MSOD Steering Group et al., (2017)
*Gender* (% females)	63.2%	56.5%
Mean age in sixth/final year	25.6 years	25.4 years
*Ethnicity*^a^ % NZ European % Maori % Pacific peoples Other	53.6% 7.4% 2.3% 36.7%	56.7% 7.5% 4.7% 40.5%

^a^Note that the MSOD report did not specify any other ethnicities aside from those listed in this table

### Study survey

An online survey was used to assess demographics (age, gender, ethnicity), year of education, dispositional factors (self-compassion, extraversion, satisfaction with life, attachment style), work burden factors (burnout, workload, patient load), and the four barriers to compassion.

#### Measuring barriers to compassion

The Barriers to Physician Compassion Questionnaire (BPCQ) is a 34-item self-report scale. Participants use a 1 (minimal) to 7 (a great deal) Likert scale to rate the extent to which each barrier prevents them from expressing compassion. Structural analyses of this instrument (Fernando & Consedine, 2014b) suggest a four-component solution corresponding to the four barriers in the Transactional Model of Physician Compassion (Fernando & Consedine, 2014a): barriers related to the physician (e.g. feeling tired and fatigued), the environment (e.g. multiple interruptions during consultations), the patient and/or their family (e.g. patient does not follow recommendations), and clinical complexity (e.g. current treatments causing unexpected adverse effects). Internal reliability in prior studies (Dev et al., 2018, 2019; Fernando & Consedine, 2017) is strong (α = 0.87–0.92) and the overall scale has adequate face, convergent, and divergent validity (Fernando & Consedine, 2014b). In this report, reliabilities for the individual barriers (α = 0.862, 0.858, 0.911, 0.888) and overall scale (α = 0.953) were good to excellent. Note that despite using a medical trainee sample, we refer to “physician-related” barriers throughout this report to maintain conceptual consistency with the original framing of the BPCQ.

#### Measuring dispositional factors

Given normative developmental changes during medical training, we assessed several dispositional factors as potential confounds. Compassion for others may be influenced by compassion for the self (Raab, 2014; Wiklund-Gustin & Wagner, 2013), hence *self-compassion* was assessed using the 12-item form of the Self-Compassion Scale (SCS-SF) (Raes et al., 2011). Total scores for the SCS-SF have good internal consistency (Raes et al., 2011), including in our sample (Cronbach’s α = 0.864).

*Extraversion*, a known predictor of compassion satisfaction amongst healthcare professionals, was assessed using the Eysenck Personality Questionnaire-Revised (EPQ-R) (Eysenck et al., 1985). Participants answer “yes” or “no” to 12 statements and the mean aggregate of the scores is used. The scale has sound reliability and concurrent validity (Eysenck et al., 1985; Francis & Pearson, 1988). Cronbach’s α = 0.861 in this report.

Because distress in medical students (including lower quality of life and well-being) impacts empathy (Neumann et al., 2011), *satisfaction with life* was assessed using the Satisfaction with Life Scale (SWLS)(Diener et al., 1985). The SWLS is a 5 item scale that uses a 1 (strongly disagree) to 7 (strongly agree) metric. The scale has high internal consistency and test–retest reliability (Diener et al., 1985), as well as convergent and discriminant validity (Pavot & Diener, 2009). Internal reliability in this report was α = 0.862.

Lastly, given that normative patterns of relating to others (termed “attachment”) can influence prosocial responses (Mikulincer et al., 2001), participants read brief descriptions of the secure, dismissive and anxious *attachment styles*, choosing the one they most identified with.

#### Measuring work burden factors

Burnout, workload, and patient load can impact empathy (Brazeau et al., 2010), compassion (Wear & Zarconi, 2008), and the barriers to care (Dev et al., 2019). Consequently, *burnout* was assessed using the Copenhagen Burnout Inventory (Kristensen et al., 2005). For this report, we used the overall burnout scale (Cronbach’s α = 0.908). Subjective workload and patient load were assessed using single item, 5-point Likert scales.

### Statistical analyses

SPSS Statistics (v.26, IBM Corporation, New York) was used to conduct all analyses. Missing data (0.3% for age, 0.6% for SCS-SF, 0.6% for SWLS and 0.3% for EPQ-R) were imputed using means. We used univariate analysis of variance (ANOVA) to test for differences in (specific) barriers to compassion by year level and evaluated the effect size of year level on each of the barriers by using ɷ^2^ statistics (ɷ^2^ = 0.01 for small effect, ɷ^2^ = 0.06 for medium effect and ɷ^2^ = 0.14 for large effect) (Lakens, 2013). We assessed whether the variance related to year-of-training was comparable across different barriers using Fisher-transformed ω^2^ statistic to conduct z-tests.

A series of two-step linear regressions evaluated the effect of year-of-training on each of the four barriers to compassion. Two-step (rather than single step) regression was chosen because it enabled us to separate out possible developmental effects on the barriers to compassion (i.e., year of training) rather than merging this variation with other demographic, dispositional, and work-burden influences. Thus, in the first step, dummy codes for Years 4 and 6 were entered (Year 5 was the referent group). In the second step, confounds (i.e. dispositional factors and work burden factors) that previously showed significant differences per year level in ANOVAs and chi-squared tests were entered. Despite the absence of gender differences per year level, gender was force entered into the regression given the established gender differences in empathy research (Smith et al., 2017). The sample was insufficiently powered to test for ethnicity as a potential confound, hence this variable was not tested. Statistical significance was set at *p* < 0.05 for all tests.

## Results

### Descriptive statistics and barriers to compassion groups analysis

63.2% of the sample were female. The sample’s mean age was 24.1 years (range 20–44 years). The sample identified primarily as New Zealand European (53.6%), followed by Chinese (11.1%), and New Zealand Māori (7.4%). ANOVAs (Table 2) showed that physician (*F*(2, 348) = 7.313*, p* = 0.001, ɷ^2^ = 0.035), environmental (*F*(2, 348) = 7.965, *p* = 0.000, ɷ^2^ = 0.038) and patient/family-related barriers (*F*(2, 348) = 8.482, *p* = 0.000, ɷ^2^ = 0.041) varied by year of training, while clinical barriers did not (*F*(2, 348) = 1.953, *p* = 0.143, ɷ^2^ = 0.005). Post-hoc means comparison tests (Table 2) revealed differences between trainees in Years 4 and 6, in physician (*p* = 0.001), environmental (*p* = 0.000), and patient/family barriers (*p* = 0.000). No significant differences were found when comparing Year 5 students with Year 4 or Year 6 students. Z-tests for Fisher-adjusted ω^2^ were not significant (*p* > 0.05), indicating that the magnitude of the association between year of training and barrier scores did not vary across the four barriers.

**Table 2:** Sample characteristics and intergroup differences by year of training among 2013–2014 New Zealand medical trainees (N = 351)

	Year 4 (n = 104)	Year 5 (n = 111)	Year 6 (n = 136)	Differences (F/χ2)	Post-hoc tests
Mean age (years) (SD)	23.1 (2.9)	23.3 (2.2)	25.6 (3.8)		
Gender: % female (n)^a^	66.3 (69)	65.8 (73)	58.8 (80)	1.877	
*Ethnicity*					
% NZ European (n)	61.5 (64)	55.0 (61)	46.3 (63)		
% Chinese (n)	7.7 (8)	11.7 (13)	13.2 (18)		
% NZ Māori (n)	8.7 (9)	7.2 (8)	6.6 (9)		
% Indian (n)	4.8 (5)	5.4 (6)	3.7 (5)		
% Pacific Island (n)	4.8 (5)	0.9 (1)	1.5 (2)		
% Korean (n)	2.9 (3)	2.7 (3)	5.1 (7)		
% Other/not stated (n)	2.9(3)	8.1 (9)	9.6 (13)		
% Filipino	1.0 (1)	0.9 (1)	0.7 (1)		
% American	0.0 (0)	0.9 (1)	2.2 (3)		
% British	1.0 (1)	1.8 (2)	1.5 (2)		
% Arab	1.9 (2)	1.8 (2)	1.5 (2)		
% South African	1.9 (1)	1.8 (2)	1.5 (2)		
% Malaysian	1.9 (2)	1.8 (2)	6.6 (9)		
Barriers to compassion, mean (SD)					
Physician	3.76 (1.37)	4.05 (1.34)	4.43(1.38)	7.313**	4 < 6**, 4 < 5, 5 < 6
Environmental	2.89(1.09)	3.19(1.01)	3.44 (1.10)	7.965***	4 < 6***, 4 < 5, 5 < 6
Patient/family	3.10(1.36)	3.52 (1.30)	3.83 (1.41)	8.482***	4 < 6***, 4 < 5, 5 < 6
Clinical	2.67(1.06)	2.92(0.98)	2.91(1.09)	1.953	4 < 5 > 6
Dispositional factors, mean (SD)					
Satisfaction with life	5.10 (1.15)	5.33 (1.22)	5.35 (1.18)	1.521	
Self-compassion	2.81 (0.70)	2.83 (0.66)	3.07 (0.69)	5.516**	4 < 5, 4 < 6*, 5 < 6*
Extraversion	1.44 (0.29)	1.40 (0.28)	1.43 (0.32)	0.368	
Attachment styles^a^					
*- % secure (n)*	58.7% (61)	58.6% (65)	62.5% (85)	0.621	
*- % dismissive (n)*	36.5% (38)	36.0% (40)	32.4% (44)		
*- % anxious (n)*	4.8% (5)	5.4% (6)	5.1% (7)		
Work burden factors, mean (SD)					
Burnout	2.60 (0.59)	2.54 (0.59)	2.47 (0.55)	1.522	
Workload^b^	3.67 (0.74)	3.39 (0.72)	3.24 (0.62)	11.696***	4 > 5**, 5 > 6,4 > 6***
Patient load	2.95 (0.74)	2.87 (0.69)	2.84 (0.66)	0.806	

Significant at *p < 0.05, **p < 0.01, ***p < 0.001

^a^indicates χ2 test; all other statistics use ANOVA F test

^b^Welch’s F used instead of ANOVA F statistic due to violation of Levene’s test of homogeneity of variances

### Identifying confounds: dispositional factors and work burden factors across year levels

ANOVA and chi-squared analyses of dispositional factors and work burden factors indicated significant differences by year level in self-compassion (*F*(2, 348) = 5.516, *p* = 0.004) and workload (adjusted *F*(2, 218.2) = 11.696, *p* = 0.000). No year of training differences were evident for satisfaction with life, extraversion, burnout, attachment styles, or gender (Table 2).

### Barriers to compassion across year levels—multivariate prediction using hierarchical regression

*Physician-related barriers*: At Step 1 (i.e. with only Year 4 and Year 6 dummy codes), the regression explained 4.0% of the variance (*F*(2,348) = 7.313, *p* = 0.001). Being a Year 6 student (*β* = 0.136, *p* = 0.028) predicted greater physician-related barriers. Introducing self-compassion, workload, and gender in Step 2 (Table 3), the model explained 15.1% variance (*F*(5,345) = 12.252, *p* = 0.000), improving goodness-of fit (*R*^2^Δ = 11.0%, *F*Δ(3,345) = 14.958, *p* = 0.000). Higher year level still predicted higher physician-related barriers (*β*_*Year 6*_ = 0.199, *p* = 0.001; *β*_*Year 4*_ = -0.136, *p* = 0.021). Higher self-compassion predicted lower barriers (*β* = -0.213, *p* = 0.000) while higher workload predicted higher barriers (*β* = 0.213, *p* = 0.000). Gender did not have a significant effect.

**Table 3:** Model summaries of 2-step multiple linear regression assessing the effect of year level on the barriers to compassion in New Zealand medical students, 2013–2014 (N = 351)

	Step 2			
	Physician	Environmental	Patient/family	Clinical
	*β* (SE)			
*Developmental effect*				
Year 4	− 0.136 (0.178)*	− 0.149 (0.144)*	− 0.139 (0.185)*	− 0.122 (0.142)^
Year 6	0.199 (0.167)**	0.155 (0.135)*	0.137 (0.174)*	0.028 (0.133)
*Dispositional factors*				
Self-compassion	− 0.213 (0.102)***	− 0.206 (0.082)***	− 0.198 (0.106)***	− 0.201 (0.081)***
*Work burden factors*				
Workload	0.213 (0.102)***	0.093 (0.083)^	− 0.009 (0.106)	0.060 (0.082)
*Gender*^b^	0.079 (0.146)	0.005 (0.118)	− 0.055 (0.152)	− 0.067 (0.117)
*Df*	5, 345	5, 345	5, 345	5, 345
R^2^	15.1%	9.7%	8.5%	5.6%
F-test	12.252***	7.414***	6.416***	4.096**

Significant at ^*p* < 0.1, *p < 0.05, **p < 0.01, ***p < 0.001 (two-tailed tests)

^b^Male coded as 1, female coded as 2

*Environmental barriers:* Step 1 explained 4.4% of the variance (*F*(2, 348) = 7.965, *p* = 0.000). Being a Year 4 student predicted lower environmental barriers to compassion (*β* = -0.129, *p* = 0.035). At Step 2 (Table 3), the model explained 9.7% of variance (*F*(5, 345) = 7.414, *p* = 0.000) and significantly improved goodness-of-fit (*R*^*2*^Δ = 5.3%, *F*Δ(3, 345) = 6.782, *p* = 0.000). Again, higher year level predicted higher barriers (*β*_*Year 6*_ = 0.155, *p* = 0.011; *β*_*Year 4*_ = -0.149, *p* = 0.014). Self-compassion also predicted lower environmental barriers (*β* = -0.206, *p* = 0.000) while workload and gender had no significant effect on environmental barriers.

*Patient/family-related barriers.* At Step 1, the model explained 4.6% of the variance (*F*(2, 348) = 8.482, *p* = 0.000). Being in Year 4 predicted lower patient/family barriers (*β* = -0.139, *p* = 0.024). At Step 2 (Table 3), the model explained 8.5% of variance (*F*(5, 345) = 6.416, *p* = 0.000) and showed improved goodness-of-fit (*R*^*2*^Δ = 3.9%, *F*Δ(3, 345) = 4.851, *p* = 0.003), with higher year level predicting higher barriers (*β*_*Year 6*_ = 0.137, *p* = 0.025; *β*_*Year 4*_ = -0.139, *p* = 0.023). Self-compassion predicted lower patient/family barriers (*β* = -0.198, *p* = 0.000) and the effects of workload and gender were not significant.

*Clinical barriers.* At Step 1, the model accounted for only 1.1% of the variance and was not statistically significant (*F*(2, 348) = 1.953, *p* = 0.143). The model showed significant improvement in goodness-of-fit at Step 2 (*R*^*2*^Δ = 4.5%, *F*Δ(3, 345) = 5.474, *p* = 0.001), accounting for 5.6% in variance (*F*(5, 345) = 4.096, *p* = 0.001)(Table 3). The effect of being a Year 4 student tended towards significance (*β* = -0.122, *p* = 0.050), however only self-compassion had a significant effect (*β* = -0.201, *p* = 0.000), once again predicting lower barriers.

## Discussion

Although compassion and empathy are distinct, analyses revealed a consistent pattern of physician, environmental, and patient (but not clinical) barriers to compassion being *greater* among more experienced medical trainees; the degree of difference was comparable across the four barriers. Such a pattern complements a small body of qualitative work examining compassion in medical students (Tierney et al., 2018; Wear & Zarconi, 2008). Moreover, it is consistent with studies of empathy decline in medical training (Hojat et al., 2009; Neumann et al., 2011), perhaps suggesting that compassion and empathy change comparably across training. The effects of training on the barriers to compassion remained even after controlling for confounds, such as gender, workload, and self-compassion.

At the same time, however, such a pattern is *inconsistent* with studies showing that the barriers to compassion are generally *lower* among more experienced and older physicians and nurses (Dev et al., 2019). The fact that barriers appear to increase across medical training but decrease thereafter suggests that changes in the barriers to compassion are not developmentally linear. Instead, they suggest the interesting possibility that there is some point in time where increasing barriers to care stop increasing and start to decline. Identifying this point and beginning to deconstruct the changes vis-à-vis care that professional experience brings would have important implications for both medical training and ongoing professional development.

As discussed in the introduction to this study, one possible explanation to seeing higher barriers to compassion during more senior years of clinical education might be explained by the process of adjustment medical students make to the clinical environment (Tavakol et al., 2012) as opposed to preconceived ideals (Gaufberg et al., 2010). In other words, it is possible that the realities of clinical work and the necessary development of internal regulatory processes (Mahoney et al., 2016; Neumann et al., 2011) lead to higher perceived (or experienced) barriers.

Alternatively, it may be that the barriers to compassion are greater in more experienced medical trainees simply because they have actually experienced them and can more accurately rate the extent to which difficulties such as burnout and fatigue (Hojat et al., 2009; Mahoney et al., 2016), negative role modelling and unsupportive work environments (Tavakol et al., 2012), or “difficult” patients (Nogueira-Martins et al., 2006) have on their ability to care. Overall, we suspect that a combination of idealism and naivety (Gaufberg et al., 2010) results in lower perceived barriers to compassion earlier in training, leading to higher perceived barriers among more senior students as they acquire a more accurate, experientially-grounded picture of clinical environments.

A second question in the current work regarded whether the different barriers to compassion varied to the same extent across training. Prior work suggested that physician, clinical, and environmental (but not patient) barriers to compassion were lower in physicians when compared to medical students (Dev et al., 2019). Considering it was clinical barriers *specifically* that did not increase across training in this medical student sample, it may be that increases in clinical knowledge help compassion to thrive. Specifically, experience may allow physicians to see clinical complexity and diagnostic/treatment uncertainty as “part and parcel” of the job, meaning that clinical factors are experienced as interfering less with the capacity to care in more experienced trainees; such a dynamic may also help explain why perceived workloads are seen as lower as students progress in training. Equally, uncertainty and complexity may place fewer demands on the more experienced student, freeing up the “headspace” (Tierney et al., 2018) required to maintain an awareness of the “person” (patient) in front of them and thus experience and show compassion. Placing these findings within the recent systematic review of compassion education by Sinclair et al. (2021), we note that clinical barriers may be moderated because existing compassion training is heavily focused on curricular learning which features compassion in certain clinical contexts. Considering the results of this study, medical school curricula might benefit from programmes that also address personal, environmental, and patient-related aspects of compassion.

Lastly, but importantly, we also observed that gender did not impact any of the four barriers to compassion assessed here. This pattern may suggest that although prosocial dispositions are broadly affected by gender, these are independent of the objective barriers that are experienced. This assumption, however, should be a subject to empirical tests.

### Study limitations

First, although the sample appears reasonably representative of the medical student population in New Zealand (New Zealand MSOD Steering Group et al., 2019), employing volunteers from two medical schools in a particular country may limit generalisability. However, empathy declines in medical training are evident globally, including in New Zealand (Lim et al., 2013), which may imply that developmental changes in prosocial processes (including barriers to compassion in general) are unlikely to differ systematically across countries. Second, data were cross-sectional hence there can be no certainty regarding any inferred developmental processes. Although we think it is unlikely, differences may reflect the peculiarities of particular cohorts of trainees rather than developmental changes; longitudinal studies are one solution to this issue, with this study serving as a foundation for further research. Third, while participation was anonymous, prosocial processes like compassion are prone to measurement bias (Fernando et al., 2017). This caveat noted, there is no particular reason to suspect differential bias across year groups, suggesting the findings should replicate. Finally, the dataset used in this study is from more than five years ago. Since the implementation of a formal “Personal and Professional Skills” curriculum during the years of data collection (i.e. 2013–2014) at the University of Auckland, there have been no major curricular changes of relevance to student compassion. Despite the age of these data, recent studies still suggest that students struggle to maintain compassion and empathy over the course of their studies due to a variety of personal, environmental and patient barriers (Laughey et al., 2021; Perrella et al., 2019; Wright et al., 2019); it appears likely that the findings in this study continue to apply in the present day.

### Concluding remarks

To our knowledge, this report represents one of the first attempts to assess possible developmental changes in a compassion-related construct (in the form of specific barriers to compassion) among clinical medical students. Our findings are in line with existing research showing declines in empathy over the course of medical training (Spatoula et al., 2019). As expected, generally, all barriers were lowest in junior and higher in more senior medical trainees. While age-related increases in barriers relating to physician (student), environmental, and patient/family-related factors were more pronounced, testing suggested little variation in the degree of difference across trainee years between different types of barriers. However, related analyses suggest that changes in the barriers to care across clinical years might be related to the development of beneficial coping skills such as self-compassion, as well as acclimatisation to the clinical environment.

Identifying how barriers to compassion—overall as well as specific barriers—vary across clinical training deepens our understanding of how compassion changes in medical trainees as well as possible reasons behind such changes. Such understanding can inform the development of targeted training and interventions to reduce the barriers to care. For example, medical education might benefit from incorporating leadership and service learning programmes, which have to date been more frequently used in nursing education, and may be helpful in addressing environmental and patient-related barriers (Sinclair et al., 2021).

Additionally, students might benefit from simply knowing that some barriers to care increase as patient contact increases or that clinical uncertainty becomes less problematic with greater clinical exposure and familiarity. Given the robust association between self-compassion and lower barriers to care (in all four barriers), and the “trainable” nature of self-compassion, interventions to enhance self-compassion in medical students are also promising. Relative to other groups, medical trainees are known to be high-achieving and perfectionistic (Enns et al., 2001), suggesting there is significant room for self-compassion to develop. Previous studies involving medical students have shown promising results for mindfulness-based interventions on compassionate helping behaviours (Fernando et al., 2017) and empathy (Barbosa et al., 2013; Bond et al., 2013) suggesting that approaches incorporating aspects of self-compassion could also have positive effects on students’ experience of the barriers to care at large.
